# Email Patient-Provider Communication and Cancer Screenings Among US Adults: Cross-sectional Study

**DOI:** 10.2196/23790

**Published:** 2021-07-30

**Authors:** Tiffany B Kindratt, Marlyn Allicock, Folefac Atem, Florence J Dallo, Bijal A Balasubramanian

**Affiliations:** 1 Public Health Program, Department of Kinesiology College of Nursing and Health Innovation University of Texas at Arlington Arlington, TX United States; 2 Department of Health Promotion and Behavioral Sciences School of Public Health Dallas UTHealth, The University of Texas Health Science Center at Houston Dallas, TX United States; 3 Center for Health Promotion and Prevention Research Harold C. Simmons Comprehensive Cancer Center University of Texas Southwestern Medical Center Dallas, TX United States; 4 Department of Biostatistics and Data Science School of Public Health Dallas UTHealth, The University of Texas Health Science Center at Houston Dallas, TX United States; 5 Department of Public and Environmental Wellness School of Health Sciences Oakland University Rochester, MI United States; 6 Department of Epidemiology, Human Genetics, and Environmental Health Sciences School of Public Health Dallas UTHealth, The University of Texas Health Science Center at Houston Dallas, TX United States

**Keywords:** email, patient-provider communication, online, patient portals, mammogram, Pap test, colon cancer screening, cancer screenings, National Health Interview Survey

## Abstract

**Background:**

The growth of electronic medical records and use of patient portals have allowed for patients and health care providers to communicate via email and direct messaging between health care visits. Email patient-provider communication (PPC) may enhance traditional face-to-face PPC by allowing patients to ask questions, receive clear explanations, engage in shared decision-making, and confirm their understanding between in-person visits. Despite increasing trends in the use of email PPC since the early 2000s, few studies have evaluated associations between email PPC and the uptake of preventive services.

**Objective:**

The objective of this study was to determine associations between the use of email PPC and the likelihood of undergoing breast, cervical, and colon cancer screenings among adults who have received health care in the past 12 months.

**Methods:**

Secondary, cross-sectional data from the 2011-2015 National Health Interview Survey were combined and analyzed. For each cancer screening, inclusion criteria were based on the age of screening recommendations and prior history of cancer diagnosis (n=35,912 for breast, n=48,512 for cervical, and n=45,884 for colon). The independent variable was whether adults used email PPC in the past 12 months (yes or no). The dependent variables were whether (1) women (aged ≥40 years) received a mammogram in the past 12 months; (2) women (aged 21-65 years) received a Pap test in the past 12 months; and (3) individuals (aged ≥50 years) received a colon cancer screening in the past 12 months. Bivariate and multivariable logistic regression analyses were conducted.

**Results:**

Adults who reported receiving all three cancer screenings in the past 12 months were more likely to be non-Hispanic White; be married or living with a partner; have a bachelor’s degree or higher education level; have health insurance coverage; and perceive their health as excellent, very good, or good (all *P*<.001). Men were more likely to receive colon cancer screenings than women (*P*<.001). Multivariable logistic regression models showed women who used email to communicate with their health care providers had greater odds of receiving breast (odds ratio [OR] 1.32, 95% CI 1.20-1.44) and cervical (OR 1.11, 95% CI 1.02-1.20) cancer screenings than women who did not use email PPC. Adults who used email to communicate with their health care providers had 1.55 times greater odds (95% CI 1.42-1.69) of receiving a colon cancer screening than those who did not use email PPC.

**Conclusions:**

Our results demonstrate that email PPC is a marker of increased likelihood of adults completing age-appropriate cancer screenings, particularly breast, cervical, and colon cancer screenings. More research is needed to examine other factors related to the reasons for and quality of email PPC between patients and health care providers and determine avenues for health education and intervention to further explore this association.

## Introduction

Email patient-provider communication (PPC) is broadly defined to include computer-based PPC “within a contractual relationship in which the health care provider has taken an active measure of responsibility for the clients” [1]. Email PPC includes messages sent through electronic personal health management tools and patient portals, which can enhance traditional face-to-face communication between health care providers and patients. Email PPC has been used by patients for asynchronous inquiries about nonacute issues, medication information, administration questions, and lab results [2]. Adults who used email PPC reported it was most useful for managing appointments, asking administrative questions, reviewing test results, requesting prescriptive refills, and asking health-related questions [2,3]. Individuals have also reported benefits of email PPC for communicating sensitive issues that patients may be too embarrassed to discuss during face-to-face encounters and follow-up visits for chronic diseases [4]. Although some studies have demonstrated that email PPC can increase the quality and efficiency of health care delivery [5], others have found that the use of electronic and email PPC, and the use of “e-visits,” may lead to more office visits and limit health care providers’ abilities to treat new patients [6]. Despite patients expressing interest in communicating with their provider via email, the uptake remains low [2]. In 2003, only 4% of adults reported using email to communicate with their health care provider. By 2018, the prevalence of email PPC had increased to 36% [7]. Among older adults, its prevalence rose from 2.7% in 2009 to 14.2% in 2018 [8]. Regardless of increasing trends, email PPC remains underutilized, and disparities exist based on demographic, socioeconomic, and health-related characteristics [7,9-12]. Previous studies have found that adults who engage in email PPC are more likely to be female and younger aged; living in urban areas; have higher levels of education and income; and have a history of chronic disease [7,9-11]. Several studies have found that non-Hispanic White adults are more likely to use email PPC than non-Hispanic Black and Hispanic adults [4], yet the usage of email PCC among Hispanic adults varies by US- and foreign-born subgroups [12]. These differences may be due to structural barriers that exist with broadband internet access, which may be more limited in poor neighborhoods [13]. Individuals with diabetes, cardiovascular disease, hypertension, a history of cancer, and multiple chronic conditions are more likely to use email PPC than adults with no chronic diseases [10,11]. Other studies have demonstrated that gay and bisexual men are more likely to use email PPC than heterosexual men [14].

Although previous research has demonstrated that quality face-to-face PPC during traditional visits is a marker of an increased likelihood of adults receiving cancer screenings and immunizations [15-20], few studies have evaluated the impact of email PPC between visits on the individual’s use of preventive services. Interventions using electronic patient health records and patient portals that engage patients in taking an active role in their health care through electronic means have been effective at improving delivery of recommended cancer screenings [21]. However, less is known about how this engagement through electronic modes of communication with providers between visits can improve cancer screening outcomes. Huang and colleagues [22] demonstrated that adults who used patient portals to schedule appointments, request referrals or prescription refills, share medical records, or communicate with health care professionals by email were more likely to receive a blood pressure check, lipid level check, influenza vaccination, or colon cancer screening than those who did not use patient portals. Totzkay and colleagues [23] found that women who used electronic medical records were more likely to receive breast cancer screenings. Despite positive findings, these studies did not directly evaluate associations between email PPC and likelihood of adults receiving immunizations and cancer screenings.

To begin to fill this gap in our understanding of how email PPC may be a marker of increased likelihood of cancer screenings, this study is part of a program of research that utilizes national health surveys to examine how predisposing and enabling factors are associated with morbidity, mortality, and use of health services. Patient experiences, including the use of face-to-face, email, and other electronic communications with health care providers, are examined as enabling factors of health services use as an extension of Anderson’s Behavioral Model of Health Services [24]. Two preliminary studies were conducted to determine whether email PPC is a marker of increased likelihood of vaccinations and cancer screenings. Using cross-sectional data from the National Health Interview Survey (NHIS), we found that adults who used email PPC had greater odds of reporting receipt of an influenza vaccine [25]. Using cross-sectional data from the Health Information National Trends Survey (HINTS), we found no difference in the odds of reporting receipt of breast, cervical, or colon cancer screening among adults who used email PPC compared to those who did not use email PPC [15]. Although nationally representative, the HINTS sample is much smaller (ie, N=3865 adults in HINTS 5 Cycle 3) than the NHIS sample (ie, N= 31,997 adults in 2019), and the focus of the survey content is geared toward cancer risk communication [26,27]. More research is needed to confirm these findings by using larger nationally representative samples focused on broader topics of morbidity, mortality, and the use of preventive services. To further explore this relation, this study aimed to determine the association between email PPC use and the likelihood of adults receiving breast, cervical, and colon cancer screenings before and after controlling for potential covariates.

## Methods

### Data Source

We analyzed secondary, cross-sectional data from the 2011-2015 NHIS. Since 1957, the NHIS has collected information on demographics, socioeconomics, and a wide range of health topics among the civilian noninstitutionalized US population [28]. During 2011-2015, the NHIS used a multistage sampling design to monitor national trends in health, illness, and disability while tracking progress toward national goals by using a computer-assisted personal interviewing system during face-to-face interviews [28]. The sampling design oversampled Hispanic, Asian, and non-Hispanic Black persons to increase the precision of estimates among racial and ethnic minorities [28]. Information about health information technology use and cancer screening behaviors have been measured annually since 2011 [29-31]. Further details of the NHIS sampling design and data collection methods have been reported previously [28].

### Participants

We limited our sample to individuals who received primary health care in the past 12 months and were within the recommended age groups for each cancer screening of interest. For breast cancer screening, for instance, our sample was limited to women aged 40 years and above without any prior history of breast cancer based on American College of Obstetricians and Gynecologists (ACOG) screening recommendations (n=35,912) [32]. For cervical cancer screenings, our sample was limited to women aged 21-65 years without any prior history of cervical cancer based on the American Cancer Society (ACS), ACOG, and the United States Preventive Services Task Force (USPSTF) screening recommendations (n=48,512) [33-35]. For colon cancer screenings, our sample was limited to adults aged 50 years and older without any prior history of colon cancer based on the ACS screening recommendations (n=45,884) [33]. Individuals who reported that they do not use the internet were excluded.

### Variables

#### Independent Variable

The NHIS measured email PPC by asking individuals, “During the past 12 months, have you ever used computers for any of the following,” specifically to “…communicate with a health care provider by email” (yes or no question) [28].

#### Dependent Variables

For breast cancer screenings, the NHIS asked women aged 30 years and older, “Have you had a mammogram during the past 12 months?” For cervical cancer screenings, women aged 18 years and older were asked, “Have you had a pap smear or pap test during the past 12 months?” Adults aged 40 years and older were asked, “During the past 12 months, have you had any test done for colon cancer [28]?” Dichotomous variables (yes or no) were created for each screening measure based on the abovementioned inclusion criteria for age.

#### Covariates

We evaluated the following covariates based on previous studies [15,25]: age (ie, 21-29 years and 30-39 years for cervical cancer screenings only, 40-49 years for breast and cervical cancer screenings only, 50-59 years, 60-70 years [60-65 years for cervical cancer screenings], or 70 years and older); sex (ie, female or male); race or ethnicity (ie, non-Hispanic White, Hispanic, non-Hispanic Black, or non-Hispanic “other” race); nativity status (born in the United States or not born in the United States); marital status (ie, never married; married or living as married; divorced, widowed, or separated); highest level of education achieved (ie, no degree, high school degree or General Educational Development tests, some college or associate degree, or bachelor’s degree or higher); insurance coverage (ie, insured or uninsured); perceived health status (ie, excellent, very good, or good vs fair or poor); and survey year.

### Statistical Analysis

Bivariate analyses were used to describe the association between demographic, socioeconomic, health-related characteristics, use of email PPC, and receipt of each cancer screening in the past 12 months (chi-square test; =.05). We calculated age-adjusted prevalence estimates of receiving breast, cervical, and colon cancer screenings among adults who reported using email PPC by using estimated marginal (least-squares) means. Crude and multivariable logistic regression procedures were used to test for associations between email PPC (independent variable) and whether they received breast, cervical, and colon cancer screenings (dependent variable) before and after controlling for covariates. Purposeful selection methods were used for building fitted multivariable logistic regression models [36]. Our multivariable models were adjusted for age (reference: youngest age group, 40-49 years for breast cancer screenings, 21-30 years for cervical cancer screenings, and 50-59 years for colon cancer screenings); race or ethnicity (reference: non-Hispanic White); marital status (reference: never married); education (reference: bachelor’s degree or higher); health insurance (reference: covered); and perceived health status (reference: fair or poor). For colon cancer screenings only, multivariable models were adjusted for sex (reference: men).

### Sensitivity Analysis

Sensitivity analyses were conducted to align with different age cutoffs for screening recommendations from other agencies based on previous research [37]. For breast cancer and colon cancer screenings, we limited the sample to women aged 50-75 years based on USPSTF recommendations [37,38]. No sensitivity analyses were conducted for cervical cancer screenings.

Data were analyzed using SAS software (version 9.4) survey procedures to account for primary sampling units, clustering, and the sophisticated weighting in the sampling design. The annual sample adult weight was divided by five to account for combining 5 years of data based on NHIS analytic recommendations [39].

This study was deemed exempt from human subjects review by the Committee for the Protection of Human Subjects at the University of Texas Health Science Center at Houston.

## Results

### Selected Characteristics

The mean ages of women who received breast and cervical cancer screenings in the past 12 months were 56.90 years (95% CI 56.71-57.08) and 41.2 (95% CI 41.00-41.40) years, respectively. The mean age of adults who received a colon cancer screening the past 12 months was 62.1 (95% CI 61.94-62.35) years. Women who reported having a breast cancer screening in the past 12 months were more likely to be non-Hispanic White; be married or living with a partner; have a bachelor’s degree or higher level of education; have health insurance coverage; and perceive their health as excellent, very good, or good (all *P*<.05). Similar results were observed for cervical cancer screenings. Additionally, US-born women (27,006/31,977, 85.5% weighted) were more likely to receive a cervical cancer screening than foreign-born women (4971/31,977, 14.5% weighted; *P*<.001). Furthermore, over half (5587/11,713, 50.9% weighted) of the adults who received a colon cancer screening were male (*P*<.001). Results were similar to those for breast and cervical cancer screenings for race or ethnicity, marital status, education, and health insurance coverage (all *P*<.001). Further details of the bivariate analyses (unweighted frequencies and weighted percentages) are reported in Table 1.

Age-adjusted prevalence estimates of receiving a breast, cervical, or colon cancer screening based on email PPC use are reported in Table 2. Among adults who received primary health care in the last 12 months, the age-adjusted prevalence of receiving a colon cancer screening was the lowest (34.4%) among those who used email PPC compared to women who underwent breast (70.5%) and cervical (70.6%) cancer screenings.

**Table 1:** Selected characteristics by receiving a breast, cervical and colon cancer screening in the last 12 months, National Health Interview Survey 2011-2015.

Characteristic		Breast cancer screening (n=35,912), n (weighted %)					Cervical cancer screening (n=48,512), n (weighted %)							Colon cancer screening (n=45,884), n (weighted %)				
		No (n=13,557)	Yes (n=22,355)	*P* value		No (n=16,530)		Yes (n=31,982)		*P* value		No (n=34,171)			Yes (n=11,713)		*P* value	
**Race or ethnicity**					.005						<.001							<.001
	Non-Hispanic White	9659 (76.3)	15,865 (76.4)			11,087 (72.0)		19,598 (68.7)				26,330 (81.6)			8476 (78.5)			
	Hispanic	1367 (8.4)	2200 (7.9)			2175 (11.3)		4801 (11.9)				2494 (6.1)			950 (6.4)			
	Non-Hispanic Black	1752 (9.6)	3121 (10.6)			2013 (9.5)		5493 (13.6)				3646 (7.6)			1698 (10.5)			
	Non-Hispanic Asian or Other	779 (5.7)	1169 (5.0)			1255 (7.1)		2090 (5.8)				1701 (4.7)			589 (4.6)			
**Nativity status**					.46						.001							0.03
	Foreign-born	1856 (13.9)	3089 (13.5)			2649 (15.9)		4971 (14.5)				3895 (11.5)			1431 (12.6)			
	US-born	11,697 (86.1)	19,263 (86.5)			13,875 (84.1)		27,006 (85.5)				30,269 (88.5)			10,279 (87.4)			
**Marital status**					<.001						<.001							<.001
	Never married	1502 (8.1)	2165 (6.6)			3498 (18.2)		7557 (18.5)				2963 (5.9)			949 (5.5)			
	Married or living with partner	6340 (61.1)	12,227 (69.2)			8752 (63.6)		17,838 (67.5)				18,594 (69.3)			6792 (72.5)			
	Divorced, widowed, or separated	5664 (30.8)	7899 (24.2)			4232 (18.2)		6507 (14.1)				12,522 (24.8)			3947 (22.0)			
**Education**					<.001						<.001							<.001
	Less than high-school graduate	1426 (9.1)	1515 (5.6)			1352 (7.5)		2076 (5.4)				2989 (7.3)			955 (6.8)			
	High-school graduate	3360 (25.2)	4857 (22.2)			3598 (22.5)		5724 (18.1)				8134 (24.0)			2468 (20.9)			
	Some college	4758 (34.7)	7325 (31.8)			6136 (36.9)		11,050 (33.8)				10,822 (30.9)			3808 (31.7)			
	Bachelor’s degree or higher	3966 (31.0)	8600 (40.3)			5400 (33.2)		13,081 (42.8)				12,127 (37.8)			4447 (40.6)			
**Health insurance**					<.001						<.001							<.001
	Not covered	1506 (11.0)	865 (3.5)			2391 (13.7)		2989 (8.0)				1882 (5.1)			302 (2.4)			
	Covered	12,016 (89.0)	21,446 (96.5)			14,078 (86.3)		28,915 (92.0)				32,223 (94.9)			11,396 (97.6)			
**Perceived health status**					<.001						<.001							.08
	Fair or poor	2760 (18.5)	2736 (11.0)			2694 (15.0)		2806 (7.7)				5765 (15.3)			2105 (16.1)			
	Excellent, very good, or good	10,792 (81.5)	19,606 (89.0)			13,831 (85.0)		29,162 (92.3)				28,385 (84.7)			9606 (83.9)			
**Survey year**					<.001						<.001							0.02
	2011	3875 (27.9)	5414 (23.8)			3493 (21.2)		7455 (23.0)				9299 (26.6)			3139 (25.8)			
	2012	1991 (15.2)	3872 (17.7)			2683 (16.3)		6222 (18.9)				5259 (16.0)			1889 (16.3)			
	2013	2323 (17.5)	4168 (19.1)			3175 (19.4)		6219 (19.6)				5899 (17.8)			2159 (19.5)			
	2014	2675 (18.9)	4554 (19.4)			3645 (21.0)		6319 (18.9)				6901 (19.3)			2260 (18.3)			
	2015	2693 (20.4)	4347 (19.9)			3534 (22.1)		5767 (19.5)				6813 (20.4)			2266 (20.1)			

**Table 2:** Age-adjusted prevalence of screenings by email patient-provider communication (PPC), National Health Interview Survey 2011-2015.

Email PPC	Breast cancer screening, OR^a^ (95% CI)	Cervical cancer screening, OR (95% CI)	Colorectal cancer screening, OR (95% CI)
No	62 (61-63)	67 (66-67)	25 (24-26)
Yes	71 (69-72)	71 (69-72)	34 (33-36)

^a^OR: odds ratio.

### Logistic Regression Analysis

Crude and adjusted logistic regression results are reported in Table 3. In adjusted models, women who used email to communicate with their health care providers had 1.32 times greater odds (95% CI 1.20-1.44) of receiving a breast cancer screening and 1.11 times greater odds (95% CI 1.02-1.20) of receiving a cervical cancer screening than women who did not use email PPC. Adults who used email to communicate with their health care providers had 1.55 times greater odds (95% CI 1.42-1.69) of receiving a colon cancer screening than those who did not use email PPC. Specific estimates for covariates included in our logistic regression models are provided in Table S1 of Multimedia Appendix 1

**Table 3:** Crude and adjusted logistic regression models, National Health Interview Survey 2011-2015.

Email PPC^a^	Breast cancer screening^b^, OR^c^ (95% CI)		Cervical cancer screening^b^, OR (95% CI)			Colorectal cancer screening^b^, OR (95% CI)	
	Crude	Adjusted	Crude	Adjusted	Crude		Adjusted
No	1.00	1.00	1.00	1.00	1.00		1.00
Yes	1.50 (1.38, 1.62)	1.32 (1.20, 1.44)	1.17 (1.08, 1.27)	1.11 (1.02, 1.20)	1.58 (1.44, 1.73)		1.55 (1.42, 1.69)

^a^PPC: patient-provider communication.

^b^For each cancer screening, multivariable models adjusted for age (reference: youngest age group, 40-49 years for breast cancer screening, 21-30 years for cervical cancer screening, 50-59 years for colon cancer screening); race or ethnicity (reference: non-Hispanic White); marital status (reference: never married); education (reference: bachelor’s degree or higher); health insurance (reference: covered); and perceived health status (reference: fair or poor). For colon cancer screening only, multivariable models adjusted for sex (reference: men).

^c^OR: odds ratio.

### Sensitivity Analysis

Crude and adjusted logistic regression results from our sensitivity analysis are reported in Table S2 of Multimedia Appendix 1. The results were similar to our analytical sample. All 95% CIs overlapped with our initial findings.

## Discussion

### Principal Findings

We aimed to determine the association between email PPC and whether adults received breast, cervical, and colon cancer screenings. Overall, we found that adults who used email to communicate with their health care providers between visits had greater odds of receiving each of the three types of screenings. These findings go beyond our previous research that used other nationally representative data sources (Medical Expenditure Panel Survey and HINTS), which demonstrated that quality face-to-face PPC increased the likelihood of adults receiving cancer screenings [15,16]. Nevertheless, there was no difference in breast, cervical, or colon cancer screening uptake among adults who did and those who did not use email PPC.

Using the NHIS, we were able to further explore the role of email PPC as a marker of the likelihood of adults receiving cancer screening using a nationally representative sample larger than that used in previous studies. For breast cancer screening, we found that women who used email PPC had 32% increased odds of receiving a mammogram compared to women who did not use email PPC. Other studies exploring whether online PPC and general health information technology use were associated with breast cancer screening found that electronic medical record and patient portal use increased women’s likelihood of receiving mammograms [23,40-42]. Moreover, we found that email PPC increased women’s odds of receiving a Pap test by 11%. This finding result differs from our study using HINTS data, which did not find any association between email PPC and cervical cancer screenings [15]. To our knowledge, only one other study has demonstrated that general electronic medical record use can increase cervical cancer screenings [41]. Finally, we found that the use of email PPC increased the likelihood of adults receiving a colon cancer screening by 55%. Our previous study using HINTS data indicated that adults had 39% higher odds of receiving a colon cancer screening; however, the results did not reach statistical significance (95% CI 0.99-1.95). Similar to studies evaluating breast cancer screening, previous research has demonstrated that adults who used patient portals to schedule appointments, request referrals or prescription refills, view decision aids, share medical records, or communicate with health care professionals by email were more likely to receive colon cancer screenings than those who did not use patient portals [22,42]. For all cancer screenings, the lack of research providing direct comparisons to our results may be due to limitations of examining email PPC only, which excludes other online communicative functions such as text messaging, mobile apps, and social media [43].

For all cancer screenings, several factors may have contributed to obtaining results different from our previous study using HINTS data [15]. The greatest factor may be the way email PPC was measured. The NHIS measured whether adults used computers or the internet to communicate with their health care provider by email [28]. A similar measure was used in the HINTS 4 survey during Cycles 1 and 3 [44]. Adults who responded “yes” on either survey may have regarded automatic emails for appointment reminders or diagnostic test results as email PPC versus directly emailing their health care provider about specific health concerns. During HINTS Cycle 3 and 4, this question was revised to directly assess whether adults exchanged health information with their health care provider via email [44]. The reasoning for and quality of communication remained unmeasured by both surveys. Future iterations of these data sources should be revised to fully capture communication behaviors to further explore the implications of email PPC on the uptake of preventive services.

### Strengths and Limitations

A strength of this study was the use of survey data from multiple years of the NHIS, a nationally representative survey that has consistently measured health behaviors, preventive health services, and a wide array of other health-related characteristics to meet national health objectives for over 60 years [28]. The depth and breadth of demographic, socioeconomic, and health-related characteristics measured by the NHIS on an annual basis allowed us to explore and control for multiple covariates in our logistic regression models. However, the NHIS does not collect characteristics on patient engagement in health care outside from assessing adults’ use of health information technology to look up health information, refill prescriptions, schedule appointments, use online chat groups, and communicate with health care providers via email that may result in unresolved confounding. Previous research has demonstrated that adults who use electronic methods of communication with their health care providers and adults who follow recommended cancer screening guidelines are more engaged in their health care than those who do not [5]. In our study, email PPC and cancer screening behaviors were both measured in the past 12 months based on how the questions were asked by the NHIS. Our results may suffer from temporality biases, as we were unable to determine whether email PPC occurred before or after receiving any cancer screening. By limiting our cancer screening outcome to the past 12 months instead of based on adherence (eg, past 2 years for mammogram, past 3 years for Pap testing, and past 10 years for colonoscopy), we minimized this potential bias. A limitation to our measurement of email PPC was that we were unable to determine the direction of the email (ie, patient to provider vs provider to patient) and whether the content of the communication was screening related or pertaining to any medical information. The clinical significance of our results may be limited due to reporting odds ratios and marginal means. The use of other marginal effects may have improved our study’s practical relevance [45]. Our cancer screening measures were self-reported. Some studies have cautioned that results from self-reported nationally representative studies may overestimate cancer screening uptake [46], whereas others have found that self-reported responses are consistent with findings from hospital records [47,48]. It is also important to note that the NHIS is a cross-sectional survey, so our results only represent associations instead of causal relations.

### Conclusions

This study begins to fill the gap in our understanding of how email PPC and direct electronic messaging between appointments may be a marker of the increased likelihood of adults receiving preventive health services, in particular, cancer screening uptake. More research is needed to determine the need for and effectiveness of targeted strategies for promoting appropriately timed cancer screenings by using web-based PPC tools such as email and direct messaging. Furthermore, there is a need for more research to examine reasons for and quality of email PPC for making preventive health care decisions.

## Appendix

Multimedia Appendix 1 Supplementary tables presenting results from logistic regression models.

## Abbreviations

ACS: American Cancer Society
ACOG: American College of Obstetricians and Gynecologists
HINTS: Health Information National Trends Survey
NHIS: National Health Interview Survey
OR: odds ratio
PPC: patient-provider communication
USPSTF: United States Preventive Services Task Force

## References

[1] Kane B, Sands DZ (1998). Guidelines for the clinical use of electronic mail with patients. The AMIA Internet Working Group, Task Force on Guidelines for the Use of Clinic-Patient Electronic Mail. J Am Med Inform Assoc.

[2] Ye J, Rust G, Fry-Johnson Y, Strothers H (2010). E-mail in patient-provider communication: a systematic review. Patient Educ Couns.

[3] Haun JN, Patel NR, Lind JD, Antinori N (2015). Large-scale survey findings inform patients' experiences in using secure messaging to engage in patient-provider communication and self-care management: a quantitative assessment. J Med Internet Res.

[4] Atherton H, Sawmynaden P, Sheikh A, Majeed A, Car J (2012). Email for clinical communication between patients/caregivers and healthcare professionals. Cochrane Database Syst Rev.

[5] Antoun J (2016). Electronic mail communication between physicians and patients: a review of challenges and opportunities. Fam Pract.

[6] Bavafa H, Hitt LM, Terwiesch C (2018). The impact of e-visits on visit frequencies and patient health: evidence from primary care. Manage Sci.

[7] Senft N, Butler E, Everson J (2019). Growing disparities in patient-provider messaging: trend analysis before and after supportive policy. J Med Internet Res.

[8] Hung L, Lyons JG, Wu C (2020). Health information technology use among older adults in the United States, 2009-2018. Curr Med Res Opin.

[9] Tarver WL, Menser T, Hesse BW, Johnson TJ, Beckjord E, Ford EW, Huerta TR (2018). Growth dynamics of patient-provider internet communication: trend analysis using the Health Information National Trends Survey (2003 to 2013). J Med Internet Res.

[10] Asan O, Cooper Ii F, Nagavally S, Walker RJ, Williams JS, Ozieh MN, Egede LE (2018). Preferences for health information technologies among us adults: analysis of the Health Information National Trends Survey. J Med Internet Res.

[11] Zhang Y, Lauche R, Sibbritt D, Olaniran B, Cook R, Adams J (2017). Comparison of health information technology use between American adults with and without chronic health conditions: findings from the National Health Interview Survey 2012. J Med Internet Res.

[12] Gonzalez M, Sanders-Jackson A, Wright T (2019). Web-based health information technology: access among Latinos varies by subgroup affiliation. J Med Internet Res.

[13] Perzynski AT, Roach MJ, Shick S, Callahan B, Gunzler D, Cebul R, Kaelber DC, Huml A, Thornton JD, Einstadter D (2017). Patient portals and broadband internet inequality. J Am Med Inform Assoc.

[14] Dahlhamer JM, Galinsky AM, Joestl SS, Ward BW (2017). sexual orientation and health information technology use: a nationally representative study of U.S. adults. LGBT Health.

[15] Kindratt T, Atem F, Dallo F, Allicock M, Balasubramanian B (2020). The influence of patient-provider communication on cancer screening. J Patient Exp.

[16] Kindratt TB, Dallo FJ, Allicock M, Atem F, Balasubramanian BA (2020). The influence of patient-provider communication on cancer screenings differs among racial and ethnic groups. Prev Med Rep.

[17] Villani J, Mortensen K (2013). Patient-provider communication and timely receipt of preventive services. Prev Med.

[18] Carcaise-Edinboro P, Bradley CJ (2008). Influence of patient-provider communication on colorectal cancer screening. Med Care.

[19] Underhill ML, Kiviniemi MT (2012). The association of perceived provider-patient communication and relationship quality with colorectal cancer screening. Health Educ Behav.

[20] Ho MY, Lai JY, Cheung WY (2011). The influence of physicians on colorectal cancer screening behavior. Cancer Causes Control.

[21] Krist AH, Woolf SH, Rothemich SF, Johnson RE, Peele JE, Cunningham TD, Longo DR, Bello GA, Matzke GR (2012). Interactive preventive health record to enhance delivery of recommended care: a randomized trial. Ann Fam Med.

[22] Huang J, Chen Y, Landis JR, Mahoney KB (2019). Difference between users and nonusers of a patient portal in health behaviors and outcomes: retrospective cohort study. J Med Internet Res.

[23] Totzkay D, Silk KJ, Sheff SE (2017). The effect of electronic health record use and patient-centered communication on cancer screening behavior: an analysis of the Health Information National Trends Survey. J Health Commun.

[24] Andersen RM (2008). National health surveys and the behavioral model of health services use. Med Care.

[25] Kindratt T, Callender L, Cobbaert M, Wondrack J, Bandiera F, Salvo D (2019). Health information technology use and influenza vaccine uptake among US adults. Int J Med Inform.

[26] Health Information National Trends Survey, Public Use Dataset. National Cancer Institute.

[27] National Health Interview Survey, 2019. Centers for Disease Control and Prevention.

[28] National Health Interview Survey – NHIS Data, Questionnaires and Related Documentation. National Center for Health Statistics, Centers for Disease Control and Prevention.

[29] Sandefer RH, Khairat SS, Pieczkiewicz DS, Speedie SM (2015). Using publicly available data to characterize consumers use of email to communicate with healthcare providers. Stud Health Technol Inform.

[30] Cohen RA, Adams PF (2011). Use of the internet for health information: United States, 2009. NCHS Data Brief.

[31] Bhandari N, Shi Y, Jung K (2014). Seeking health information online: does limited healthcare access matter?. J Am Med Inform Assoc.

[32] (2017). Practice Bulletin Number 179: breast cancer risk assessment and screening in average-risk women. Obstet Gynecol.

[33] Smith RA, Andrews KS, Brooks D, Fedewa SA, Manassaram-Baptiste D, Saslow D, Brawley OW, Wender RC (2017). Cancer screening in the United States, 2017: a review of current American Cancer Society guidelines and current issues in cancer screening. CA Cancer J Clin.

[34] (2016). Practice Bulletin No. 168: cervical cancer screening and prevention. Obstet Gynecol.

[35] Curry SJ, Krist AH, Owens DK, Barry MJ, Caughey AB, Davidson KW, Doubeni CA, Epling JW, Kemper AR, Kubik M, Landefeld CS, Mangione CM, Phipps MG, Silverstein M, Simon MA, Tseng C, Wong JB, US Preventive Services Task Force (2018). Screening for cervical cancer: US Preventive Services Task Force recommendation statement. JAMA.

[36] Hosmer DW, Lemeshow S, Sturdivant RX (2013). Applied Logistic Regression, 3rd Edition.

[37] Siu AL, U.S. Preventive Services Task Force (2016). Screening for breast cancer: U.S. Preventive Services Task Force recommendation statement. Ann Intern Med.

[38] Bibbins-Domingo K, Grossman DC, Curry SJ, Davidson KW, Epling JW, García FAR, Gillman MW, Harper Diane M, Kemper Alex R, Krist Alex H, Kurth Ann E, Landefeld C Seth, Mangione Carol M, Owens Douglas K, Phillips William R, Phipps Maureen G, Pignone Michael P, Siu Albert L, US Preventive Services Task Force (2016). Screening for colorectal cancer: US Preventive Services Task Force recommendation statement. JAMA.

[39] (2016). Variance Estimation Guidance, NHIS 2006-2015.

[40] Henry SL, Shen E, Ahuja A, Gould MK, Kanter MH (2016). The online personal action plan: a tool to transform patient-enabled preventive and chronic care. Am J Prev Med.

[41] Thompson CA, Gomez SL, Chan A, Chan JK, McClellan SR, Chung S, Olson C, Nimbal V, Palaniappan LP (2014). Patient and provider characteristics associated with colorectal, breast, and cervical cancer screening among Asian Americans. Cancer Epidemiol Biomarkers Prev.

[42] Krist AH, Woolf SH, Hochheimer C, Sabo RT, Kashiri P, Jones RM, Lafata JE, Etz RS, Tu S (2017). Harnessing information technology to inform patients facing routine decisions: cancer screening as a test case. Ann Fam Med.

[43] Jiang S, Street RL (2017). Factors influencing communication with doctors via the internet: a cross-sectional analysis of 2014 HINTS Survey. Health Commun.

[44] Survey Instruments – Health Information National Trends Survey. National Cancer Institute.

[45] Norton EC, Dowd BE, Maciejewski ML (2019). Marginal effects-quantifying the effect of changes in risk factors in logistic regression models. JAMA.

[46] Rauscher GH, Johnson TP, Cho YI, Walk JA (2008). Accuracy of self-reported cancer-screening histories: a meta-analysis. Cancer Epidemiol Biomarkers Prev.

[47] Barratt A, Cockburn J, Smith D, Redman S (2000). Reliability and validity of women's recall of mammographic screening. Aust N Z J Public Health.

[48] King ES, Rimer BK, Trock B, Balshem A, Engstrom P (1990). How valid are mammography self-reports?. Am J Public Health.

