# Supplementary material for: Email Patient-Provider Communication and Cancer Screenings Among US Adults: Cross-sectional Study
Source: JMIR Cancer. 2021 Jul 30;7(3):e23790. doi: 10.2196/23790 (PMC8367146; doi:10.2196/23790)
Supplement: Multimedia Appendix 1 [file cancer_v7i3e23790_app1.docx]

**Table S1. Crude and adjusted^a^ logistic regression model results with covariates, NHIS 2011-2015.**

|  | **Crude** | **Adjusted^a^** |
| --- | --- | --- |
|  | **OR (95% CI)** | **OR (95% CI)** |
| **Breast Cancer Screening** |  |  |
| **E-mail PPC** |  |  |
| No | 1.00 | 1.00 |
| Yes | 1.50 (1.38-1.62) | 1.32 (1.20-1.44) |
| **Race/Ethnicity** |  |  |
| Non-Hispanic White |  | 1.00 |
| Hispanic |  | 1.33 (1.20-1.47) |
| Non-Hispanic Black |  | 1.42 (1.31-1.53) |
| Non-Hispanic Asian/Other |  | 0.91 (0.79-1.04) |
| **Marital Status** |  |  |
| Never Married |  | 1.00 |
| Married/Live with partner |  | 1.38 (1.25-1.52) |
| Divorced/Widow/Separated |  | 1.01 (0.91-1.12) |
| **Education** |  |  |
| Bachelor’s degree/higher |  | 1.00 |
| Some college |  | 0.77 (0.71-0.82) |
| HS graduate |  | 0.77 (0.71-0.83) |
| Less than HS graduate |  | 0.64 (0.57-0.72) |
| **Health Insurance** |  |  |
| Covered |  | 1.00 |
| Not Covered |  | 0.33 (0.29-0.37) |
| **Perceived Health Status** |  |  |
| Fair/Poor |  | 1.00 |
| Excellent/Very good/Good |  | 1.65 (1.52-1.80) |
| **Survey Year** |  |  |
| 2015 |  | 1.00 |
| 2014 |  | 1.08 (0.98-1.18) |
| 2013 |  | 1.20 (1.09-1.31) |
| 2012 |  | 1.28 (1.17, 1.41) |
| 2011 |  | 1.04 (0.95-1.13) |
| **Cervical Cancer Screening** |  |  |
| **E-mail PPC** |  |  |
| No | 1.00 | 1.00 |
| Yes | 1.17 (1.08-1.27) | 1.11 (1.02-1.20) |
| **Race/Ethnicity** |  |  |
| Non-Hispanic White |  | 1.00 |
| Hispanic |  | 1.21 (1.11-1.32) |
| Non-Hispanic Black |  | 1.74 (1.61-1.90) |
| Non-Hispanic Asian/Other |  | 0.75 (0.68-0.83) |
| **Marital Status** |  |  |
| Never Married |  | 1.00 |
| Married/Live with partner |  | 1.39 (1.30-1.50) |
| Divorced/Widow/Separated |  | 1.25 (1.15-1.36) |
| **Education** |  |  |
| Bachelor’s degree/higher |  | 1.00 |
| Some college |  | 0.74 (0.69-0.79) |
| HS graduate |  | 0.69 (0.64-0.74) |
| Less than HS graduate |  | 0.63 (0.56-0.71) |
| **Health Insurance** |  |  |
| Covered |  | 1.00 |
| Not Covered |  | 0.51 (0.47-0.56) |
| **Perceived Health Status** |  |  |
| Fair/Poor |  | 1.00 |
| Excellent/Very good/Good |  | 1.76 (1.63-1.90) |
| **Survey Year** |  |  |
| 2015 |  | 1.00 |
| 2014 |  | 1.41 (0.96-1.13) |
| 2013 |  | 1.21 (1.11-1.31) |
| 2012 |  | 1.39 (1.28-1.51) |
| 2011 |  | 1.41 (1.30-1.52) |
| **Colon Cancer Screening** |  |  |
| **E-mail PPC** |  |  |
| No | 1.00 | 1.00 |
| Yes | 1.58 (1.44-1.73) | 1.55 (1.42-1.69) |
| **Sex** |  |  |
| Female |  | 1.00 |
| Male |  | 1.38 (1.30-1.47) |
| **Race/Ethnicity** |  |  |
| Non-Hispanic White |  | 1.00 |
| Hispanic |  | 1.17 (1.04-1.31) |
| Non-Hispanic Black |  | 1.55 (1.41-1.69) |
| Non-Hispanic Asian/Other |  | 1.02 (0.90-1.17) |
| **Marital Status** |  |  |
| Never Married |  | 1.00 |
| Married/Live with partner |  | 1.14 (1.02-1.26) |
| Divorced/Widow/Separated |  | 1.04 (0.94-1.16) |
| **Education** |  |  |
| Bachelor’s degree/higher |  | 1.00 |
| Some college |  | 1.00 (0.94-1.06) |
| HS graduate |  | 0.88 (0.82-0.95) |
| Less than HS graduate |  | 0.94 (0.84-1.05) |
| **Health Insurance** |  |  |
| Covered |  | 1.00 |
| Not Covered |  | 0.46 (0.39-0.55) |
| **Perceived Health Status** |  |  |
| Fair/Poor |  | 1.00 |
| Excellent/Very good/Good |  | 0.91 (0.85-0.98) |
| **Survey Year** |  |  |
| 2015 |  | 1.00 |
| 2014 |  | 0.98 (0.89-1.08) |
| 2013 |  | 1.16 (1.06-1.27) |
| 2012 |  | 1.09 (1.00-1.18) |
| 2011 |  | 1.07 (0.98-1.17) |

*^a^Abbreviations: CI=confidence interval; NHIS=National Health Interview Survey; OR=odds ratio.*

**Table S2. Sensitivity analysis for crude and adjusted^a^ logistic regression models based on USPSTF screening recommendations (ages 50-75 years), NHIS 2011-2015.**

|  | **Crude** | **Adjusted^a^** |
| --- | --- | --- |
|  | **OR (95% CI)** | **OR (95% CI)** |
| **Breast Cancer Screening** |  |  |
| **E-mail PPC** |  |  |
| No | 1.00 | 1.00 |
| Yes | 1.74 (1.55-1.94) | 1.47 (1.31-1.65) |
| **Race/Ethnicity** |  |  |
| Non-Hispanic White |  | 1.00 |
| Hispanic |  | 1.36 (1.19-1.55) |
| Non-Hispanic Black |  | 1.41 (1.29-1.54) |
| Non-Hispanic Asian/Other |  | 0.89 (0.74-1.06) |
| **Marital Status** |  |  |
| Never Married |  | 1.00 |
| Married/Live with partner |  | 1.35 (1.19-1.54) |
| Divorced/Widow/Separated |  | 0.94 (0.83-1.08) |
| **Education** |  |  |
| Bachelor’s degree/higher |  | 1.00 |
| Some college |  | 0.77 (0.70-0.84) |
| HS graduate |  | 0.74 (0.67-0.82) |
| Less than HS graduate |  | 0.58 (0.50-0.67) |
| **Health Insurance** |  |  |
| Covered |  | 1.00 |
| Not Covered |  | 0.30 (0.25-0.35) |
| **Perceived Health Status** |  |  |
| Fair/Poor |  | 1.00 |
| Excellent/Very good/Good |  | 1.69 (1.54-1.85) |
| **Survey Year** |  |  |
| 2015 |  | 1.00 |
| 2014 |  | 1.16 (1.03-1.29) |
| 2013 |  | 1.24 (1.11-1.39) |
| 2012 |  | 1..37 (1.23-1.53) |
| 2011 |  | 1.06 (0.96-1.18) |
| **Colon Cancer Screening** |  |  |
| **E-mail PPC** |  |  |
| No | 1.00 | 1.00 |
| Yes | 1.58 (1.44-1.73) | 1.57 (1.43-1.72) |
| **Sex** |  |  |
| Female |  | 1.00 |
| Male |  | 1.39 (1.31-1.48) |
| **Race/Ethnicity** |  |  |
| Non-Hispanic White |  | 1.00 |
| Hispanic |  | 1.17 (1.04-1.31) |
| Non-Hispanic Black |  | 1.53 (1.39-1.68) |
| Non-Hispanic Asian/Other |  | 1.03 (0.90-1.18) |
| **Marital Status** |  |  |
| Never Married |  | 1.00 |
| Married/Live with partner |  | 1.14 (1.03-1.27) |
| Divorced/Widow/Separated |  | 1.08 (0.96-1.20) |
| **Education** |  |  |
| Bachelor’s degree/higher |  | 1.00 |
| Some college |  | 1.00 (0.93-1.06) |
| HS graduate |  | 0.87 (0.81-0.94) |
| Less than HS graduate |  | 0.97 (0.86-1.10) |
| **Health Insurance** |  |  |
| Covered |  | 1.00 |
| Not Covered |  | 0.46 (0.39-0.55) |
| **Perceived Health Status** |  |  |
| Fair/Poor |  | 1.00 |
| Excellent/Very good/Good |  | 0.90 (0.83-0.98) |
| **Survey Year** |  |  |
| 2015 |  | 1.00 |
| 2014 |  | 0.96 (0.87-1.07) |
| 2013 |  | 1.16 (1.06-1.27) |
| 2012 |  | 1.08 (0.99-1.19) |
| 2011 |  | 1.08 (0.99-1.19) |

*^a^Abbreviations: CI=confidence interval; NHIS=National Health Interview Survey; OR=odds ratio; USPSTF=United States Preventive Services Task Force.*
